# Editorial: Biowaste valorization utilizing microbial systems

**DOI:** 10.3389/fmicb.2023.1213598

**Published:** 2023-05-18

**Authors:** Debarati Paul, Justyna Bohacz, Shashi Kant Bhatia

**Affiliations:** ^1^Amity Institute of Biotechnology, Amity University Uttar Pradesh (AUUP), Noida, India; ^2^Department of Environmental Microbiology, Faculty of Agrobioengineering, University of Life Sciences in Lublin, Lublin, Poland; ^3^Department of Biological Engineering, College of Engineering, Konkuk University, Seoul, Republic of Korea; ^4^Institute for Ubiquitous Information Technology and Application, Konkuk University, Seoul, Republic of Korea

**Keywords:** biowaste, valorization, fertilizer, biofuel, pigment

The demand for energy resources and chemicals is continuously increasing around the globe due to the ever-increasing population and industrialization (Sinha et al., 2021; Kang et al., 2023). The use of microbial cell factories is getting attention to valorize waste into valuable products (biofuel, fertilizers, pigments, enzymes, etc.) due to its eco-friendly nature and ability to generate revenue simultaneously (Bohacz et al., 2020; Vinayak et al., 2021; Paul et al., 2023). Optimization of bioprocesses depending on the organisms' nutritional requirement, cultivation conditions, and involvement of cellular metabolic pathways leads to enhanced production of value-added commodities (Bhatia et al., 2023). Microbes also have the potential to decompose complex organic waste into simpler molecules that can be used as fertilizers to improve plant growth and productivity. This Research Topic is focused on the valorization of waste into valuable products utilizing microbial systems and four articles were published (Figure 1).

**Figure 1:**
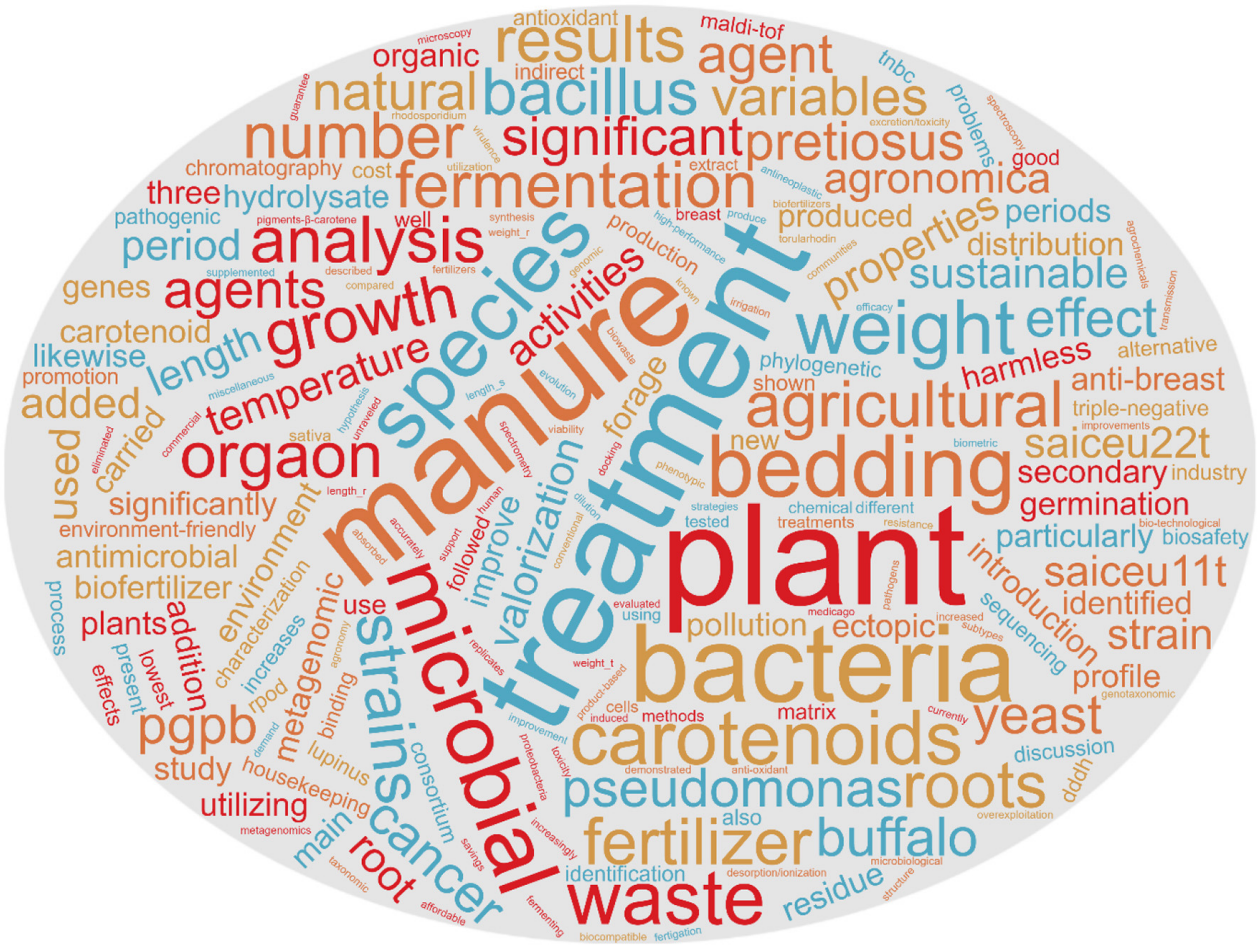
Word cloud of keywords used in the Research Topic (common words displayed with a larger font).

The first article discusses the use of “mandi” waste to produce carotenoids from oleaginous red yeast *Rhodosporidium* sp. for cancer treatment. The carotenoid extract was composed of β-carotene, torulene, and torularhodin and demonstrated antioxidant, antimicrobial, and anti-breast cancer activities. *In silico* analysis showed good binding energy toward VEGF receptors. Overall the process is sustainable and eco-friendly for carotenoid production from red yeast having anti-breast cancer activities (Sinha et al.).

The second article studied the growth of *Lupinus albus* using an organic fertilizer matrix (ORGAON^®^) made from horticultural waste, with the addition of two bacterial strains (*Bacillus pretiosus* and *Pseudomonas agronomica*). Results revealed that both bacterial strains added individually to the ORGAON^®^ and sterile ORGAON^®^ (ORGAON^®^st) organic matrix improved certain plant biometric variables. *B. pretiosus* enhanced root weight, total weight, plant length, and the number of secondary roots, while *P. agronomica* improved root length and the number of secondary roots. Genotaxonomic analysis confirmed the suitability of both strains for improving crop yield in fields. Biosafety tests were conducted and yielded positive results (Mora, Fernández Pastrana, Gutiérrez Oliva et al.). The use of chemical fertilizers pollutes soil and groundwater, while agri-food industry waste endangers the environment and human health. To counteract this, natural biofertilizers are made from agricultural waste via microbiological treatments by breaking down the waste into simple molecules. Biosafety is ensured by metagenomic analysis to exclude pathogens. A study characterizes a biofertilizer from agricultural waste and tests the addition of Plant Growth Promoting Bacteria (PGPB) *P. agronomica* and *B. pretiosus*, individually and in a consortium. Eubacterial and archaebacterial strains have been identified and proven to be non-pathogenic. The biofertilizer significantly stimulates *Mendicago sativa* growth (Mora, Fernández Pastrana, Probanza Lobo et al.). The fourth article assessed the effects of different microbial agents on buffalo manure bedding treatment. The study analyzed temperature, humidity, pH, and microbial distribution. Four agents had a harmless effect, but agent F was the most efficient and cost-effective. The ectopic fermentation bedding treatment process was divided into three periods, with a heating period above 75°C that degraded numerous harmful bacteria. The study provides guidance for manure-resource utilization in cattle farms and mitigating its harmful effects (Niu et al.).

From the content of articles published in this Research Topic, it can be concluded that the application of various types of waste as feedstock for microbial culture for producing carotenoids or biofertilizers, and treatment of buffalo manure to obtain bedding, are promising approaches for a sustainable future. These studies highlight the potential of utilizing microbial systems for waste management and revenue generation, while also promoting environmental sustainability and human health.

## Author contributions

SB drafted the Editorial while DP and JB contributed to editing. All authors conceived and designed the work and provided final approval of the version to be published.

## References

[B1] Bhatia S. K. Rajesh Banu J. Singh V. Kumar G. Yang Y.-H. (2023). Algal biomass to biohydrogen: Pretreatment, influencing factors, and conversion strategies. Bioresour. Technol. 368, 128332. 10.1016/j.biortech.2022.128332 36414137

[B2] Bohacz J. Mozejko M. Kitowski I. (2020). *Arthroderma tuberculatum* and *Arthroderma multifidum* isolated from soils in rook (*Corvus frugilegus*) colonies as producers of keratinolytic enzymes and mineral forms of N and S. Int. J. Environ. Res. Public. Health. 17, 9162. 10.3390/ijerph17249162 33302453 PMC7763491

[B3] Kang B.-J. Jeon J.-M. Bhatia S. K. Kim D.-H. Yang Y.-H. Jung S.. (2023). Two-stage bio-hydrogen and polyhydroxyalkanoate production: Upcycling of spent coffee grounds. Polymers 15, 681. 10.3390/polym15030681 36771983 PMC9919241

[B4] Paul D. Kumari P. K. Siddiqui N. (2023). Yeast carotenoids: Cost-effective fermentation strategies for health care applications. Fermentation 9, 147. 10.3390/fermentation9020147

[B5] Sinha S. Singh G. Arora A. Paul D. (2021). Carotenoid production by red yeast isolates grown in agricultural and “Mandi” waste. Waste Biomass Valorizat. 12, 3939–3949. 10.1007/s12649-020-01288-8

[B6] Vinayak V. Khan M. J. Varjani S. Saratale G. D. Saratale R. G. Bhatia S. K. (2021). Microbial fuel cells for remediation of environmental pollutants and value addition: Special focus on coupling diatom microbial fuel cells with photocatalytic and photoelectric fuel cells. J. Biotechnol. 338, 5–19. 10.1016/j.jbiotec.2021.07.003 34245783

